# Essential oil of *Pterodon polygalaeflorus* Benth attenuates nociception in mice

**DOI:** 10.1590/1414-431X20187356

**Published:** 2018-10-04

**Authors:** A.N. Coelho-de-Souza, C.F. dos-Santos, L.N. Lopes-Filho, F.R. Holanda, A.C. Oliveira, Y.A. Gomes-Vasconcelos, K.A. Oliveira, F.W. Ferreira-da-Silva, K.S. Silva-Alves, J.H. Leal-Cardoso

**Affiliations:** 1 Laboratório de Fisiologia Experimental, Instituto Superior de Ciências Biomédicas, Universidade Estadual do Ceará, Campus do Itaperi, Fortaleza, CE, Brasil; 2 Centro Universitário Christus, Fortaleza, CE, Brasil; 3 Curso de Engenharia Civil, Centro de Ciências Exatas e Tecnologia, Universidade Estadual Vale do Acaraú, Campus CIDAO, Sobral, CE, Brasil

**Keywords:** Essential oil, Pterodon polygalaeflorus, Antinociception, Anti-inflammatory activities, Beta-elemene, Caryophyllene

## Abstract

Essential oils (EO) are volatile liquids responsible for the aroma of plants. *Pterodon polygalaeflorus* seeds have received widespread use in folk medicine for the treatment of inflammatory diseases. For this reason and because *Pterodon polygalaeflorus* seeds have great EO content, which is frequently pharmacologically active, the present study aimed to evaluate the antinociceptive effect of EO from *Pterodon polygalaeflorus* (EOPPgfl) and its acute toxic effects. The EEOPPgfl sample, which was extracted by steam distillation of the seeds, had a yield of 2.4% of the seeds weight and had, as major constituents, beta-elemene (48.19%), trans-caryophyllene (19.51%), and epi-bicyclosesquiphellandrene (12.24%). The EOPPgfl sample showed mild acute toxicity and its calculated median lethal dose (LD_50_) was 3.38 g/kg. EOPPgfl (20–60 mg/kg) showed antinociceptive activity as evidenced by several tests and inhibited writhing induced by acetic acid. The maximum effect was obtained with the 30 mg/kg dose and at 60 min after its administration. EOPPgfl also decreased formalin-induced nociception, as verified by the inhibition of the first and second phase of the formalin test. At 30 mg/kg, EOPPgfl also decreased thermally stimulated nociception. Nociception may be related to inflammatory and antiedematogenic activity and at doses ranging 10–100 mg/kg, EOPPgfl blocked dextran- and carrageenan-induced edema. The results demonstrated that EOPPgfl presented, at doses approximately 100 times smaller than LD_50_, an antinociceptive effect that probably was due to anti-inflammatory activities.

## Introduction

Aromatic formulations have been used throughout human history for spiritual, social, and medicinal purposes. For medicinal purposes, they are not usually used as essential oils but mainly as infusions, teas, and incense (1).

Essential oils (EO) are volatile and oleaginous liquids with usually pleasant aromas that provide odor to plants (2). EO of aromatic plants from Northeast Brazil have been studied from the chemical (3,4) and pharmacologic point of view (1,3,5,6) showing great potential for therapeutic use. Northeastern flora is abundant in aromatic species, whose EO consist of a mix of compounds, mostly phenylpropanoic and terpene derivatives. This chemical complexity allows EO to have several biological effects, such as antiparasitic (7), antimicrobial (8), analgesic and anti-inflammatory (9), diuretic and hypotensive (10), antimalarial (11), gastro-protector (1,6), cell excitability inhibitor (12–14), among others.

*Pterodon polygalaeflorus* Benth or *Commilobium polygalaeflorus*, popularly known as “sucupira branca” (white sucupira), is a tree common in the Brazilian Northeast region and abundant in Maranhão and Piauí states (15,16). It has great regional importance because of the widespread use of its shell and seeds in popular medicine as hydroalcoholic infusions for the treatment of chronic diarrhea (16), pharyngitis (15), and chronic pain and inflammation, such as in arthritis (17) and rheumatic diseases.

The chemical composition and pharmacological effects of the essential oil of *Pterodon polygalaeflorus* Benth (EOPPgfl) seeds have been previously investigated (15,16). It has demonstrated antispasmodic action on tracheal smooth muscle by blockade of voltage-operated calcium channels (15) and on intestinal smooth muscle by myogenic mechanisms, mainly mediated through an intracellular mechanism (16).

Due to its popularity in the treatment of inflammatory diseases, its previously observed pharmacological activity, and the great EO yield of the seeds, the present study aimed to evaluate the antinociceptive effect of a new sample of EOPPgfl in experimental models of pain and the acute toxic effects in order to seek new substances with therapeutic potential.

## Material and Methods

### Extraction and chemical analysis of EOPPgfl

EOPPgfl was extracted from the seed of *P. polygalaeflorus* Benth collected in the municipality of São Raimundo das Mangabeiras, Maranhão state in February 1999 by steam distillation method. Chemical composition was determined by gas chromatography coupled to mass spectrometry in the Technological Development Park of Federal University of Ceará (PADETEC-UFC) by Craveiro (18), as previously described. Briefly the conditions were as follows: Hewlett-Packard 6971 (USA); dimethylpolysiloxane DB-1 fused silica capillary column (30 m × 0.25 mm; 0.1 μm); helium (1 mL/min) as carrier gas; injector temperature: 250°C; detector temperature: 200°C; column temperature: 35–180°C at 4°C/min and then 180-250°C at 10°C/min; and mass spectra: electronic impact 70 eV. The compounds were identified using a NIST mass spectral library search (more information on https://chemdata.nist.gov/).

### Drugs

All drugs were of analytical purity. Dextran, carrageenan, histamine, and serotonin were from Sigma Chemical Company (USA). Cyproheptadine, formaldehyde, acetic acid, indomethacin, morphine, and naloxone were from Cinética Química Ltda. (Brazil).

Solutions were prepared by adding the pure substance to sterile saline (0.9% NaCl w/v). EOPPgfl was prepared in sterile saline, containing Tween 80, 0.1% v/v, followed by automatic stirring. After homogenization, the solution was administered by the orogastric route.

### Animals

This study complied with the ethical principles for animal research. Swiss mice (25–30 g) from the Central Vivarium of Federal University of Ceará were kept under constant temperature (22±2°C) with a 12 h light/12 h dark cycle and free access to food and water in the Laboratory of Electrophysiology of Excitable Tissue 1–3 days prior to the experiment. The study was done from 2000 to 2002.

### Assessment of acute toxic effects

The evaluation of acute toxic effects was done by determination of the median lethal dose (LD_50_) and observation of changes in behavioral parameters.

To determine LD_50_, the animals were randomly separated into 6 groups: 1 control and 5 experimental groups. Each experimental group received, through orogastric cannula, different concentrations of EOPPgfl (1–8 g/kg). The control group received only vehicle (0.1% Tween 80 in 0.9% saline). The animals were then placed in cages with water and feed *ad libitum* and observed for a period of 72 h. After this period, the number of deaths in each group was observed and reported as percentage of the total number of animals. Calculation of LD_50_ was done by semi-logarithmic interpolation, where the EOPPgfl doses were plotted on the abscissa axis and the values corresponding to the probit percentage of deaths on the ordinate axis.

To evaluate changes in behavioral parameters, animals were randomly separated in 5 experimental groups (10 animals/group). The animals were initially observed for 72 h for the following behavioral parameters: alertness, analgesia, spontaneous motor activity, locomotion, sedation, response to touch, nasal secretion, piloerection, ptosis, dyspnea, urination, diarrhea, and seizure. They were then treated with the same doses of EOPPgfl or vehicle and observed for the same parameters during the first 3 h and, at 24-h intervals, for 72 h.

### Antinociceptive activity

To evaluate the EOPPgfl anti-nociceptive activity, the acetic acid-induced writhing test (19), the paw-licking response to the formalin test (20), and the hot plate test (21) were used.

For the writhing test, different groups of animals (n = 8–10 animals/group) received EOPPgfl (10–100 mg/kg, orally) or vehicle (0.1% Tween 80 in sterile saline, orally), 60 min before, or indomethacin (10 mg/kg, *ip*), 30 min before noxious stimulation with *ip* injection of acetic acid (0.6% v/v; 0.1 mL/10 g body weight). Then, abdominal writhes were recorded for 20 min, starting 10 min after acetic acid injection. In another experimental series, a time course for the effect of the oil (30 mg/kg, orally) administered 15, 30, 60, and 120 min before noxious stimulation was carried out. Antinociceptive activity is reported as the number of abdominal constrictions.

For the formalin test, different groups of animals received EOPPgfl (10-100 mg/kg) orally or vehicle, 60 min before the treatment with 20 µL of formalin (2.5% v/v) administered into the sub-plantar region of the right hind paw. The time the animal spent licking the paw during the first 5 min (early phase) and from 15 to 30 min (late phase) post-formalin injection served as measures of sensitivity. The test was done at ambient temperature of 22–26°C and care was taken to exclude environmental disturbance (high temperature, noise, and excessive movement) that might interfere with the animal's response. In another experimental series, a group of animals received naloxone (2 mg/kg, *sc*), an opioid antagonist, 15 min before treatment with oil (60 mg/kg orally) or morphine (5 mg/kg, *sc*). After 30 min of morphine administration, the formalin test was performed.

For the hot plate test, a mouse was placed on a plate maintained at 50.0 ± 1°C and the latency of its reaction to this nociceptive stimulus (number of seconds before it licked its hind paw or jumped) was quantified, with an interruption time ≤45 s. The test used only mice that in a pretest showed a hot plate reaction time ≤20 s. The latency of the reaction to nociception was measured at time 0 (60 min after EOPPgfl, 30 mg/kg, or vehicle administration) and then at 30 min intervals up to the 180th min.

### Antiedematogenic activity

Antiedematogenic activity of EOPPgfl was evaluated by the carrageenan-, dextran-, or formalin-induced paw edema test.

In order to obtain carrageenan- and dextran-induced paw edema, the phlogistic agents (both 300 µg/paw) were injected into the sub-plantar region of the right hind paw. The contralateral paw received an equal volume of sterile saline. The animals received EOPPgfl (10–60 mg/kg) orally or vehicle. Indomethacin (10 mg/kg, *ip*) or cyproheptadine (10 mg/kg, *ip*) were used as positive controls. The time course of the carrageenan-induced paw edema was considered to have three phases: a first (from 1 to 90 min), a second (from 91 to 150 min), and a third phase (from 151 to 240 min) (22). For formalin-induced paw edema, the same animals submitted to the nociception test were used. In these animals, formalin (20 µL of 2.5% formalin) was injected *sc* into the right hind paw. The animals received EOPPgfl (60 mg/kg) orally or vehicle. Paw licking was then measured during the first 5 min (considered as the first phase of formalin test) and at 15 min after the formalin injection (considered as the second phase of the test).

Paw edema volumes for antiedematogenic activity tests were measured with a plethysmometer (Panlab, S.L.U., Digital Water Plethysmometer Le 7500, Spain). Edema was considered to be the difference in volume between two paws, among the different time-periods and time zero.

### Statistical analysis

All data are reported as means±SE, where n is the number of experiments. The difference between two groups was assessed by the unpaired Student's *t*-test. The significance (P<0.05) of the results that demanded multiple comparisons was initiated with the Kolmogorov-Smirnov test for normality of data distribution. In the cases in which normality was rejected, Kruskal-Wallis one-way ANOVA on ranks followed by Dunn's *post hoc* test was done; otherwise parametric one-way ANOVA followed by Bonferroni or Holm-Sidak *post hoc* test was employed. For each experimental series analyzed and parametric data, the ANOVA parameters d.f._1_ (degree of freedom related to the number of independent variables, between groups), d.f._2_ (degree of freedom related to the number of observations in each group, within group), and F (ratio of the mean square variation estimates of data between groups and within group) were used. For non-parametric data, ANOVA on rank parameters d.f. (degree of freedom) and H (value computed by ranking all observations from smallest to largest without regard to treatment group) was used. P<0.05 was considered significant for all *post hoc* tests. Statistical analysis was done using Sigma Stat and Sigma Plot software (Systat Inc., USA).

## Results

### Extraction and chemical analysis of EOPPgfl

The oil extracted by steam distillation of seeds gave a yield of 2.4% weight. In the oil sample, seven sesquiterpenes (with % of oil weight) were identified: beta-elemene (48.19%), trans-caryophyllene (19.51%), epi-bicyclosesquiphellandrene (12.24%), gamma-elemene (4.44%), alpha-copaene (4.13%), alpha-humulene (3.32%), alloaromadendrene (2.45%); and two alcohols: (3.17%) spatulenol and (2.55%) farnesol.

### Toxic effect of EOPPgfl

As can be seen in Table 1, no death occurred in animals treated with doses of EOPPgfl below or equal to 3 g/kg. However, at doses above 3 g/kg, toxic signs such as lethargy, motor incoordination, tachypnea, ptosis, and asphyxia were observed and the calculated LD_50_ was 3.38 g/kg.

**Table 1:** Symptoms and deaths of animals treated with essential oil of *Pterodon polygalaeflorus* (EOPPgfl).

Group	Dose (mg/kg)	Deaths	Symptoms
Control	-	0	none
EOPPgfl	1000	0	none
	2000	0	none
	3000	0	Lethargy, motor incoordination and anesthesia
	4000	n=5 (62.5%)	Lethargy, motor incoordination and anesthesia
	8000	n=5 (62.5%)	Motor incoordination, tachypnea and asphyxia

n=8 animals for control and each dose.

### Antinociceptive activity

**Figure 1:**
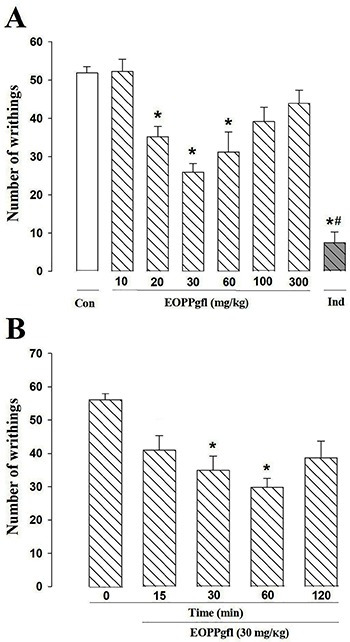
Effect of essential oil of *Pterodon polygalaeflorus* (EOPPgfl) on acetic acid-induced writhing. *A*, Effect of several doses (10–300 mg/kg, orally) of EOPPgfl and of indomethacin (Ind, 10 mg/kg, *ip*) on the number of writhings; EOPPgfl and Ind were administered 60 and 30 min, respectively, before acetic acid administration. Ten min after acetic acid administration, the writhings were counted during 20 min. Con: control. *B*, Time course of the EOPPgfl effect (30 mg/kg, orally). Each animal received a single dose of EOPPgfl. Data are reported as means±SE (n=8–10). *A*, *P<0.001 compared to control; ^#^P<0.001 compared to 30 mg/kg EOPPgfl (one-way ANOVA, d.f._1_=7, d.f._2_=60, F=17.931, followed by Holm-Sidak *post hoc* test). *B*, *P=0.002 compared to control (Kruskal-Wallis ANOVA on ranks, d.f.=4, H=16.923, followed by Dunn's *post hoc* test).

EOPPgfl (20, 30, and 60 mg/kg) and indomethacin (10 mg/kg) significantly reduced (P<0.05, ANOVA, Dunn test) writhing number from 51.9±1.62 counts (n=10) (control value) to 35.2±2.64 (n=10), 25.9±2.32 (n=10), 31.2±5.8, and 7.5±2.8 counts (n=8), respectively (Figure 1A). This reduction corresponded to 32.2, 50.1, 39.8, and 85.5%. The effect of EOPPgfl was time-dependent since EOPPgfl (30 mg/kg), administered 15, 30, 60, or 120 min prior to the time of writhing measurement, induced writhing reduction that amounted to 27.0, 37.7, 46.8, and 31.2%, respectively. The EOPPglf-induced writhing reduction is shown in Figure 1B; the reduction was significant at 30 and 60 min.

**Figure 2:**
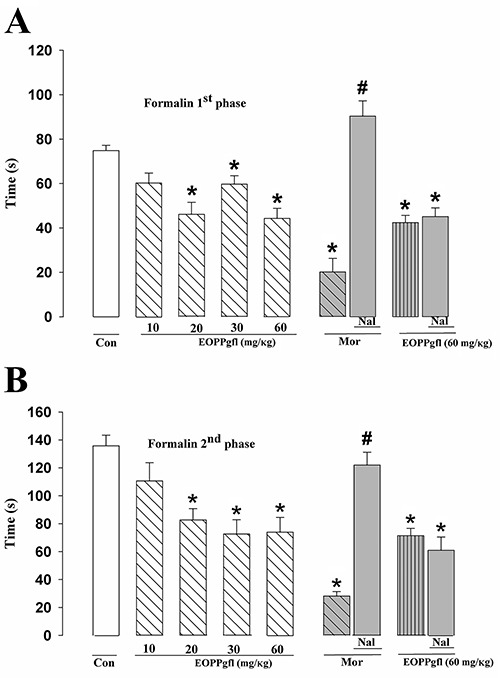
Effect of essential oil of *Pterodon polygalaeflorus* (EOPPgfl) on formalin test. Effect of several doses (10–60 mg/kg, orally) of EOPPgfl, morphine (Mor; 5 mg/kg, *sc*), and naloxone (Nal; 2 mg/kg, *sc*) on paw licking from 0 to 5 min (1st phase of formalin test; panel *A*) and from 15 to 30 min (2nd phase of formalin test; panel *B*) after formalin administration (intraplantar injection of 20 µL of 2.5% formalin (v/v) solution in distilled water). Con, control (intra-paw injection of 20 µL of saline). Data are reported as means±SE (n=8). *A*, *P<0.001 compared to control; ^#^P<0.001 compared to Mor (Kruskal-Wallis ANOVA on ranks, d.f.=8, H=43.151 followed Dunn’s *post hoc* test). *B*, *P<0.001 compared to control; ^#^P<0.001 compared to Mor (parametric one-way ANOVA, d.f._1_=8, d.f._2_=55, F=11.961 followed Bonferroni *post hoc* test).

In the formalin test, EOPPgfl (20, 30, and 60 mg/kg) significantly reduced the time the animal spent licking its paw in both phases of the test (Figure 2A and B) compared to the control (1st phase 74.85±2.26 s (n=8) and the 2nd phase 135±7.61 s (n=8)). At the dose of 20 mg/kg, for the first (Figure 2A) and second phase (Figure 2B), the time of licking was reduced to 46.1 and 82.6 s, respectively, at the dose of 30 mg/kg, to 59.7 and 72.6 s (n=7), and at the dose of 60 mg/kg, to 44.3 and 74.1 s (n=7). This effect was not altered (P>0.05, ANOVA) by subcutaneous injection of naloxone, an antagonist of opioid receptor (Figure 2A and B).

**Figure 3:**
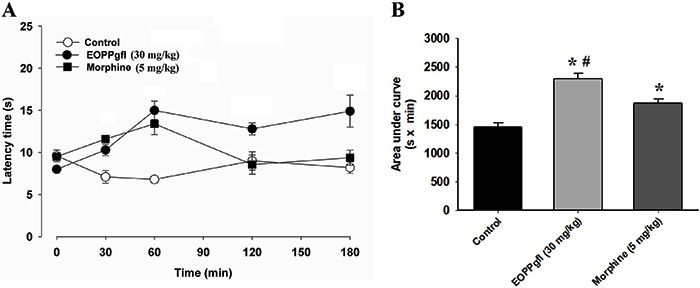
Effect of essential oil of *Pterodon polygalaeflorus* (EOPPgfl) on noxious thermal stimulation. *A*, Time course of latency of nociceptive response to paw exposure to 50±0.5°C. Data are reported as means±SE (n=8). B, Area under the curves shown in *A*. *P<0.05 compared to control; ^#^P<0.001 compared to morphine (parametric one-way ANOVA, d.f._1_=2, d.f._2_=21, F=27.191, followed Holm-Sidak *post hoc* test).

As assessed by the hot plate test, EOPPgfl significantly increased the latency time for nociceptive response to the thermal stimulus starting at 30 min and spanning throughout the observation period of 180 min. The peak of EOPPgfl effect occurred at 60 min when the latency time increased from 6.5 (control value) to 15.0 s. The effect of EOPPgfl was similar to that of morphine, although the effect of the oil lasted longer (Figure 3).

### Antiedematogenic activity

**Figure 4:**
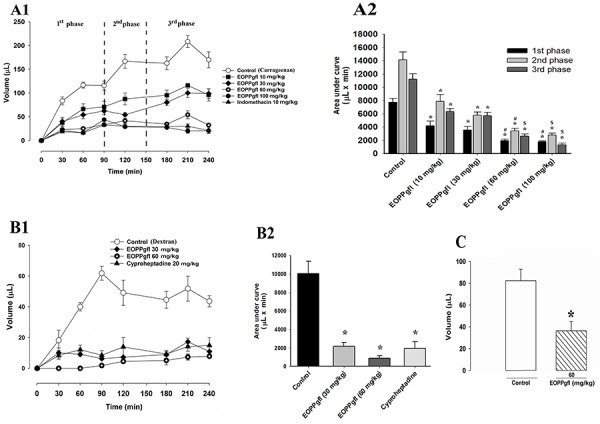
Effect of essential oil of *Pterodon polygalaeflorus* (EOPPgfl) on carrageenan-, dextran-, and formalin-induced edema. *A1*, Time course of alteration induced by EOPPgfl (orally) and indomethacin (10 mg/kg, *ip*) on edema induced by carrageenan (control, intraplantar injection of 50 μL of 6.0 mg/mL carrageenan solution in saline). *A2*, Area under the curves shown in *A1* for the 1st, 2nd, and 3rd phase of carrageenan edematogenic effect. *P<0.001 compared to control; ^#^P<0.001 compared to 10 mg/kg; ^$^P<0.001 compared to 10 and 30 mg/kg (parametric one-way ANOVA, 1st phase, d.f._1_=4, d.f._2_=35, F=23.675; 2nd phase, d.f._1_=4, d.f._2_=35, F=35.545; 3rd phase, d.f._1_=4, d.f._2_=35, F=59.183, followed by Holm-Sidak *post hoc* test). *B1*, Time course of alteration induced by EOPPgfl (orally) and cyproheptadine (10 mg/kg, *ip*) on edema induced by dextran (control, intraplantar injection of 50 μL of 6.0 mg/mL dextran solution in saline). *B2*, Area under the curves shown in *B1* for dextran edematogenic effect. *P<0.05 compared to control (parametric one-way ANOVA, d.f._1_=3, d.f._2_=28, F=28.003, followed by Holm-Sidak *post hoc* test). *C*, Alteration induced by EOPPgfl (orally) on edema induced by formalin (control, intraplantar injection of 20 μL of formalin 2.5% v/v in saline). *P=0.003, compared to control (non-paired *t*-test). Data are reported as means±SE (n=8-10 in all cases).

EOPPgfl significantly inhibited the carrageenan-induced edema from the 30th to the 240th min of the experiment (Figure 4A1 and A2). At the three phases of carrageenan-induced edema, the paw volume increase underwent inhibition with all EOPPgfl doses, as demonstrated by the decrease of the area under the curve of Figure 4A2. At doses of 100 mg/kg, the effect of EOPPgfl was similar to the effect of indomethacin (inhibition of 80%). EOPPgfl at 30 and 60 mg/kg also inhibited the edema induced by dextran (Figure 4B) and at 60 mg/kg inhibited formalin-induced edema (2.5%) (Figure 4C). In the edema induced by dextran, the effect of EOPPgfl (30 and 60 mg/kg) occurred throughout the observation period and was similar to the effect of cyproheptadine. EOPPgfl at 60 mg/kg produced a 55.7% inhibition of the edema caused by formalin.

## Discussion

This study was designed to evaluate the antinociceptive, antiedematogenic, and acute toxic effects of EOPPgfl in experimental models of pain and inflammation. The results demonstrated that EOPPgfl, with the chemical composition here described, presented very low acute toxicity and antinociceptive effects, probably due to anti-inflammatory activities.

The sample of *P. polygalaefllorus* seeds of the present investigation produced the same yield (2.4% of EO) found by Evangelista et al. (15) and Leonhardt et al (16), thus confirming the great EO yield of the seeds. It is also worth mentioning that this yield was obtained using a cheap and easy method of EO extraction: steam distillation (23). The chromatographic analysis identified 100% of the compounds, predominantly sesquiterpenes, which largely differed from that of the previous investigation (15,16). In the present case, there was a major constituent (beta-elemene, 48.2%, followed by trans-caryophyllene, 19.5%), whilst in the previous study a major constituent was not identified (β-cariophyllene, 20.6%, followed by spatulenol, 16.6%). This is not surprising, since it is known that EO compositions differ with the place and season of collection (2); the time of collection of the present and previous samples differed (February *vs* October, respectively). Since there is a large difference in chemical composition and it may affect the pharmacological results, care should be taken to obtain the appropriate EOPPgfl sample.

The results demonstrated that the calculated LD_50_ for EOPPgfl, given orally, was 3.4 g/kg allowing the oil to be classified within the category of low-toxicity essential oils (2) and to be considered a safe oil regarding acute toxic aspects. It is important to note that the doses that inhibit nociception and edema formation in the various models tested are in the range of 10–100 mg/kg, which represent doses below 3% of LD_50_. Thus, EOPPgfl can be considered to have high therapeutic potentiality. Moreover, this study also showed that EOPPgfl possesses antinociceptive activity on chemical and thermal nociception models.

In the hot plate test, 30 mg/kg EOPPgfl increased the latency time to the thermal stimulus. This test of thermal nociception is a good method for measuring the effects of opioid analgesics but it is not sensitive to the analgesic effects of nonsteroidal anti-inflammatory agents (24,25). EOPPgfl increased the response time to the thermal stimulus in the hot plate test, suggesting that EOPPgfl acted primarily at the spinal medulla and/or higher central nervous system levels (20), but not excluding an indirect mechanism. In order to investigate the participation of an indirect mechanism of the effect of EOPPgfl, its action in acetic acid-induced writhing and in the formalin tests was investigated and the oil showed antinociceptive effects in both tests.

Acetic acid-induced writing is a model sensitive to analgesic substances of central or peripheral action, allowing the assessment of varied mechanisms of action (19). Acetic acid causes the release of endogenous mediators such as PGE2 and PGF2α, histamine, bradykinin, and serotonin and stimulates the nerve endings directly by pH reduction (1). Therefore, the effectiveness of EOPPgfl in decreasing acetic acid-induced contortions, although suggesting an involvement of primarily neural mechanisms, does not exclude a antinociceptive effect of EOPPgfl due to anti-inflammatory activity, secondary to inhibitory action on the phlogistic activity of autacoids such as histamine, serotonin, and/or prostaglandin. Despite a dose-dependent relationship, EOPPgfl doses above 60 mg/kg no longer had a significant antinociceptive effect in acetic acid-induced writhing, which was surprising. Although we do not have a clear explanation for this finding, it can be suggested that in these doses, some components of EOPPgfl somehow prevent the action of the constituent promoting antinociceptive activity. Although pronounced, the effect of EOPPgfl was lower than that caused by indomethacin (10 mg/kg, *ip*), a peripherally acting drug that inhibits prostaglandin synthesis

It is known that the formalin test is a behavioral evaluation method used to measure the efficacy of antinociceptive agents with different mechanisms of action (25,26). In the first phase, starting immediately after formalin injection and with a duration of 3 to 5 min (20,27), the nociception is attributed to a peripheral neural mechanism and related to the direct chemical stimulation of type C nociceptor afferent fibers and, in part, of type Aδ fibers and is associated with the release of excitatory amino acids, nitric oxide, and substance P. In the second phase, which begins 15 to 20 min after the injection and lasts for 20 to 40 min, pain is attributed to inflammatory activity and/or alteration of central processing (25,28,29). In the formalin test, EOPPgfl was effective in both first and second phases of the test. The effectiveness of EOPPgfl during the first phase of formalin test suggests a peripheral neural mechanism. On the other hand, the effectiveness EOPPgfl during the second phase of formalin test is consistent with an anti-inflammatory action and/or alteration of central processing. In these effects, there was probably no involvement of opioid receptors since they were not blocked by naloxone (30), which blocked the effect of morphine.

Velozo et al. (31) showed that *P. polygalaeflorus* has anti-inflammatory activity by modulation of B cells and lymphocyte activation. Due to the effect of EOPPgfl in the second phase of the formalin test and because of the medicinal use of *P. polygalaeflorus* seed extracts for the treatment of inflammatory diseases, we hypothesized that EOPPgfl acted by a mechanism that includes an indirect anti-inflammatory effect. Thus, in order to investigate the participation of an anti-inflammatory mechanism of EOPPgfl, the effectiveness of EOPPgfl in paw edema induced by noxious stimulation was investigated. Formalin, carrageenan, and dextran were used as inflammatory agents. EOPPgfl inhibited edema from the three substances suggesting that its anti-nociceptive effect is primarily due to a large spectrum anti-inflammatory activity. It is important to highlight that this antiedematogenic activity was coherent with reports on the pharmacological effects of EOPPgfl major constituents. The sesquiterpene beta-elemene, in investigations with macrophages of rats, was observed to inhibit the synthesis of PGE2 and NO (32), two potent mediators of pain and inflammation (33). An essential oil with trans-caryophyllene as a major constituent has demonstrated anti-nociceptive and anti-inflammatory activity (34). Therefore, the activity of the oil can be partly explained by the fact that its main constituents present potent pharmacological effects related to the activities under study.

The investigation of the anti-inflammatory mechanism of EOPPgfl action was not the objective of this study. However, it is known that in the second phase of the formalin test, the release of inflammatory mediators occurs, and that there are differences in the edema induced by carrageenan and dextran. Dextran, within 30 min after its injection, leads to edema mediated by histamine and 5-hydroxytryptamine (serotonin) (35), which contribute with the increase of vascular permeability and fluid extravasation (36) due to degranulation of mast cells (37). Carrageenan induces an acute vascular response with protein-rich exudate and containing large numbers of neutrophils (37). The inflammatory response has three distinct phases (22). The first relates to the release of histamine and serotonin, the second (90–150 min) is mediated by the release of bradykinin (38), and finally in the third phase, the mediator is suspected to be prostaglandin (22). In the late phase of the formalin test, the release of inflammatory mediators (20) occurs such as histamine, bradykinin, and prostaglandin (39), thus the edema must be due to such mediators.

EOPPgfl inhibited formalin-induced paw edema, and with a similar potency, inhibited the dextran-induced edema (77.5%), and the first phase of carrageenan-induced edema (76.98%). This suggests that EOPPgfl may be inhibiting the synthesis, release, and/or effects of histamine and serotonin.

In conclusion, EOPPgfl was active in nociception and acute inflammation in rodents at very low doses in relation to its LD_50_, which is compatible with its use in folk medicine. Given the large yield of the seeds, it can be hypothesized that EOPPgfl is at least part of the active principle of the effects of the seeds preconized in folk medicine. Therefore, the current investigation provides information for the search of a novel candidate for the prevention and treatment of inflammation, either EOPPgfl (with a chemical composition here described) or a combination of its major constituents.
